# Metformin and SARS-CoV-2: mechanistic lessons on air pollution to weather the cytokine/thrombotic storm in COVID-19

**DOI:** 10.18632/aging.103347

**Published:** 2020-05-27

**Authors:** Javier A. Menendez

**Affiliations:** 1 Program Against Cancer Therapeutic Resistance (ProCURE), Metabolism and Cancer Group, Catalan Institute of Oncology, Girona, Spain; 2 Girona Biomedical Research Institute (IDIBGI), Girona, Spain

**Keywords:** air pollution, particulate matter, inflammation, aging, COVID-19

## Abstract

Pathological signaling in the lung induced by particulate matter (PM) air pollution partially overlaps with that provoked by COVID-19, the pandemic disease caused by infection with the novel coronavirus SARS-CoV-2. Metformin is capable of suppressing one of the molecular triggers of the proinflammatory and prothrombotic processes of urban PM air pollution, namely the mitochondrial ROS/Ca^2+^ release-activated Ca^2+^ channels (CRAC)/IL-6 cascade. Given the linkage between mitochondrial functionality, ion channels, and inflamm-aging, the ability of metformin to target mitochondrial electron transport and prevent ROS/CRAC-mediated IL-6 release might illuminate new therapeutic avenues to quell the raging of the cytokine and thrombotic-like storms that are the leading causes of COVID-19 morbidity and mortality in older people. The incorporation of infection rates, severity and lethality of SARS-CoV-2 infections as new outcomes of metformin usage in elderly populations at risk of developing severe COVID-19, together with the assessment of bronchial/serological titers of inflammatory cytokines and D-dimers, could provide a novel mechanistic basis for the consideration of metformin as a therapeutic strategy against the inflammatory and thrombotic states underlying the gerolavic traits of SARS-CoV-2 infection.

##  

### Particulate matter air pollution and SARS-CoV-2/COVID-19: A mechanistically linked pathway illuminating a therapeutic opportunity for metformin

Particulate matter (PM) air pollution concentrations frequently encountered in major cities can trigger the release of proinflammatory interleukins (e.g., IL-6) from alveolar macrophages, promoting an acceleration of arterial thrombosis [1]. Analogously, infection with the novel SARS-CoV-2 coronavirus can stimulate a *too-little-too-late* type-I interferon-mediated innate immune response, which is inherently accompanied by dysregulated secretion of IL-6 from alveolar macrophages [2, 3]. The so-called *cytokine storm* – involving overproduction of proinflammatory cytokines and overactivation of immune cells (hyperinflammation) – ultimately drives an acute respiratory distress syndrome (ARDS), one of the leading causes of mortality in patients with severe COVID-19 disease [4, 5]. Intriguingly, patients with severe COVID-19 admitted to the intensive care unit are at highest thrombotic risk, with acute pulmonary embolism being the most common thrombotic complication [6]. The ability of COVID-19 to predispose to thromboembolism, which can fuel futile cycles of hyperinflammatory responses that aggravate SARS-CoV-2 pathogenesis [7, 8], is increasingly viewed as a major factor in disease severity and mortality. It is thus not surprising that long-term exposure to PM has been recently proposed as a key contributor to COVID-19 mortality in the United States [9]. Likewise, the elevated levels of PM air pollution in Northern Italy and central Spain have been postulated as a putative risk factor underlying the extremely high COVID-19 fatality rates observed in these European regions [10–12].

The link between air pollution and COVID-19 severity can be viewed merely as the passive result of a *carrier* action of virus particles by PM; yet, one should acknowledge that PM air pollution is also a principal cause of chronic systemic and airway inflammation, ultimately leading to innate immune system hyperactivation, elevated production of proinflammatory cytokines, and thrombosis [1, 10, 13–16]. The physiopathological overlap between PM-driven inflammatory cytokine production and the cytokine/thrombotic storm in patients with COVID-19 might also suggest a *boosting* action of the former on the SARS-CoV-2 mechanism of disease (Figure 1). Therapeutically, if the chronic pulmonary effects of PM impact the prognosis of COVID-19, it then follows that small molecules with acceptable risk profiles that can block the molecular trigger(s) of IL-6 release from alveolar macrophages in response to PM might also mitigate the aggressive proinflammatory/prothrombotic nature of COVID-19. Using sophisticated cell and mouse models, a groundbreaking study by the Budinger group established that the anti-diabetic drug metformin – through its capacity to inhibit mitochondrial complex I – suppressed the mitochondrial reactive oxygen species (ROS) signaling necessary for the opening of Ca^2+^ release-activated Ca^2+^ (CRAC) channels in the generation of IL-6 from alveolar macrophages upon exposure to PM (Figure 2) [1]. Because the use a respiratory filter in people residing in areas with high levels of PM air pollution validated the causal link between PM exposure and levels of IL-6-related systemic markers [17], these findings altogether support metformin use as a preventive strategy for the mortality attributable to PM air pollution worldwide [1]. In the same line, it would be relevant to test whether metformin could suppress the cytokine and thrombotic-like storms in COVID-19 before they begin, thereby lowering the risk of severe disease in high-risk individuals.

**Figure 1:**
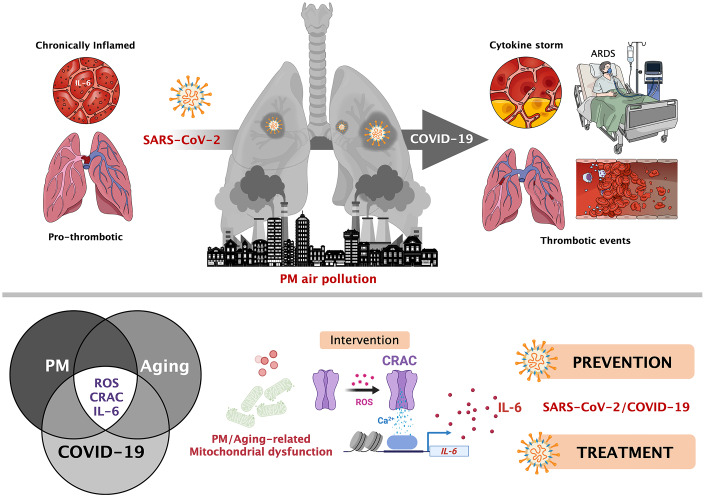
**Particulate matter air pollution and SARS-CoV-2/COVID-19: A mechanistically linked pathway illuminating a therapeutic opportunity for metformin.** *Top.* Pathological signaling in the lung induced by particulate matter (PM) air pollution partially overlaps with that caused by severe SARS-CoV-2/COVID-19, namely the release of proinflammatory interleukins (e.g., IL-6) from alveolar macrophages via mitochondrial reactive oxygen species (ROS)-driven activation of Ca^2+^ release-activated Ca^2+^ (CRAC) channels, lastly promoting an acceleration of thrombotic events. Patients already experiencing a chronic cytokine response might be at higher risk of COVID-19 lethal complications after SARS-CoV-2 infection. *Bottom*. Given the linkage between mitochondrial functionality, ion channels, and inflammation in human aging, therapeutic interventions capable of targeting mitochondrial electron transport and prevent mitochondrial ROS/CRAC-mediated IL-6 release (e.g., metformin) might illuminate a preventive/prophylactic mechanism of action to quell the raging of the cytokine and thrombotic-like storms that are the leading causes of COVID-19 morbidity and mortality in older people. In an acute scenario of SARS-CoV-2-driven hyperinflammation, small molecule CRAC channel inhibitors may also be contemplated as a means of treating patients with severe COVID-19 at risk for progressing to typical/atypical ARDS.

**Figure 2:**
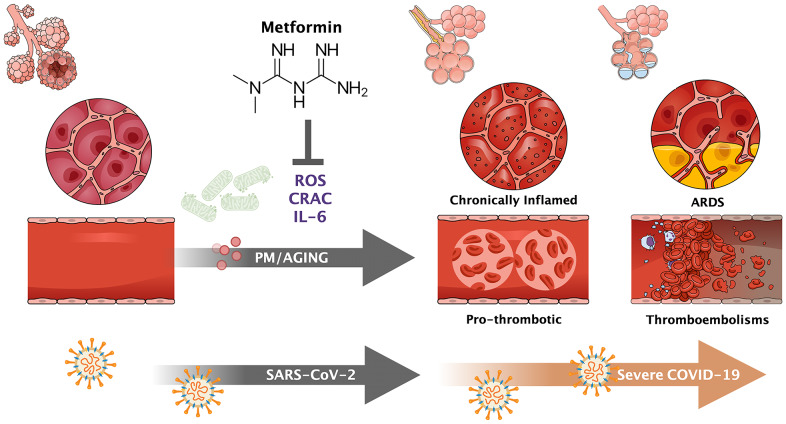
**CRAC-targeted activity of metformin: From preventive therapy of the premature death attributable to PM air pollution to geroprotector against the gerophilic and gerolavic traits of SARS-CoV-2 infection.** The ability of metformin to suppress the signaling by mitochondrial reactive oxygen species (ROS) that are necessary for the opening of Ca^2+^ release-activated Ca^2+^ channels in the generation of IL-6 from alveolar macrophages upon exposure to PM air pollution might mechanistically extend to the immune dysregulation/inflammation and thrombotic events driven by the systemic release of IL-6 from lung macrophages in response to SARS-CoV-2 infection. By restraining the raging of cytokine and thrombotic-like storms, two of the leading causes of morbidity and mortality in SARS-CoV-2 infection, metformin might be considered a putative geroprotector against the gerophilic and gerolavic traits of COVID-19 disease.

### ROS/CRAC/IL-6-targeted activity of metformin: From preventive therapy of the premature death attributable to PM air pollution to geroprotector against the gerophilic and gerolavic traits of SARS-CoV-2 infection

Severe COVID-19 illness and death is more common in people aged 60 and older with underlying conditions, which can include chronic respiratory system disease not only due to chronic exposure to PM air pollution but also to immuno-senescence and inflamm-aging phenomena [18–22]. Given the linkage between mitochondria functionality, ion channels including CRAC, and inflamm-aging [23], the ability of metformin to target mitochondrial electron transport and prevent ROS/CRAC-mediated IL-6 release might illuminate a preventive (and prophylactic) measure to quell the raging of the cytokine and thrombotic-like storms that are the leading causes of COVID-19 morbidity and mortality in older people. Such CRAC-related mechanism of action [24] capable of preventing systemically IL-6-driven thrombotic events [1], together with the multi-faceted capacity of metformin to ameliorate immunometabolism-related inflammation and alleviate ARDS [25–27] -which are believed to be the main risk factors for a worse outcome in the elderly with COVID-19 [28, 29]- could provide a novel mechanistic basis for the recently proposed geroprotective role of metformin against the *gerophilic* and *gerolavic* traits of SARS-CoV-2 infection [30].

Previous randomized clinical trials and numerous retrospective observational studies have consistently associated metformin administration with significant improvement in risk factors of aging-related diseases (cardiovascular, neurodegenerative, and cancer) beyond type 2 diabetes [31–33]. Research is now urgently needed to test whether metformin might additionally reduce the comorbidity, infection rate, severity, and lethality of SARS-CoV-2 infection, with special emphasis on the elderly risk groups accounting for the majority of severe COVID-19 disease and fatalities to date. Clinical trial strategies such as the TAME (Targeting Aging with Metformin) study, which plans to enroll 3,000 older subjects (ages 65–79) without type 2 diabetes who will be randomly assigned to 1,500 mg metformin daily or placebo for 4 years to measure time to a new occurrence of a composite outcome that includes cardiovascular events, cancer, dementia, and mortality [31, 33], provides an ideal opportunity to explore the recently proposed strategy of metformin as a low-cost geroprotector for prevention of SARS-CoV-2 [30]. In the meanwhile, observational studies in residential care nursing homes and day-care centers where older adults at significant risk of COVID-19 outbreaks are receiving metformin for treatment of type 2 diabetes (along with the assessment of bronchial/serological levels of inflammatory cytokines and markers of pro-thrombotic/hypercoagulable states such as D-dimers) might provide a deeper comprehension of how metformin can protect and potentiate common preventive strategies such as distancing measures, mask-wearing, and hand-washing.

### CRAC channels and treatment of typical/atypical ARDS in severe COVID-19

Targeting hyperinflammation in severe COVID-19 patients may be critical for reducing mortality. One might therefore wonder whether treatment with indirect (e.g., metformin [1]) or direct (e.g., CM4620 [34]) small-molecule inhibitors of CRAC channels could improve clinical outcomes in hospitalized patients with moderate/severe COVID-19. CM4620-IE, a potent and selective small molecule CRAC channel inhibitor that prevents channel overactivation and has demonstrated efficacy in patients with hypoxemia secondary to systemic inflammatory response syndrome in acute pancreatitis [34], will be trialed in patients with severe COVID-19 pneumonia at risk for progressing to ARDS (ClinicalTrials.gov identifier: NCT04345614). Because a subset of severe COVID-19 infections have a delayed onset of respiratory distress despite the severity of hypoxemia that clearly differs from classic ARDS but principally involves a catastrophic microvascular injury and thrombosis [35–37], it might be relevant to carefully evaluate the impact of targeting the mitochondrial ROS/CRAC/IL-6 signaling cascade in the respiratory, inflammatory, and survival outcomes during the “typical” and “atypical” presentation of ARDS in COVID-19 patients. Nonetheless, the findings from the clinical testing of CRAC-targeting drugs might link, at a mechanistic level, the metformin lessons on air pollution to ride (out) the cytokine/thrombotic storm in severe SARS-CoV-2/COVID-19.

## Disclaimer and Limitations

This perspective merely aims to stimulate new ideas as part of the global efforts aimed to develop new preventive/treatment strategies against the SARS-CoV-2/COVID-19 outbreak. Accordingly, this perspective does not represent medical advice or therapeutic recommendations to either COVID-19 patients or people at risk of SARS-CoV-2 infection.
